# Statistical optimization of bambara groundnut protein isolate-alginate matrix systems on survival of encapsulated *Lactobacillus rhamnosus* GG

**DOI:** 10.3934/microbiol.2017.4.713

**Published:** 2017-08-21

**Authors:** Kanyanat Kaewiad, Nattha Kaewnopparat, Damrongsak Faroongsarng, Juraithip Wungsintaweekul, Sanae Kaewnopparat

**Affiliations:** 1 Department of Pharmaceutical Technology, Faculty of Pharmaceutical Sciences, Prince of Songkla University, Hat Yai, Songkhla, 90112, Thailand; 2 Department of Pharmacognosy and Pharmaceutical Botany, Faculty of Pharmaceutical Sciences, Prince of Songkla University, Hat Yai, Songkhla, 90112, Thailand; 3 Drug Delivery System Excellence Center, Faculty of Pharmaceutical Sciences, Prince of Songkla University, Hat Yai, Songkhla, 90112, Thailand

**Keywords:** *Lactobacillus rhamnosus* GG, encapsulation, bambara groundnut protein isolate, gastrointestinal tract, storage

## Abstract

Encapsulation may protect viable probiotic cells. This study aims at the evaluation of a bambara groundnut protein isolate (BGPI)-alginate matrix designed for encapsulating a probiotic *Lactobacillus rhamnosus* GG. The response surface methodology was employed to gain the optimal concentrations of BGPI and alginate on encapsulation efficiency and survival of encapsulated cells. The capsules were prepared at the optimal combination by the traditional extrusion method composed of 8.66% w/v BGPI and 1.85% w/v alginate. The encapsulation efficiency was 97.24%, whereas the survival rates in an acidic condition and after the freeze-drying process were 95.56% and 95.20%, respectively—higher than those using either BGPI or alginate as the encapsulating agent individually. The designed capsules increased the probiotic *L. rhamnosus* GG survival relative to free cells in a simulated gastric fluid by 5.00 log cfu/ml after 3 h and in a simulated intestinal fluid by 8.06 log cfu/ml after 4 h. The shelf-life studies of the capsules over 6 months at 4 °C and 30 °C indicated that the remaining number of viable cells in a BGPI-alginate capsule was significantly higher than that of free cells in both temperatures. It was demonstrated that the BGPI-alginate capsule could be utilized as a new probiotic carrier for enhanced gastrointestinal transit and storage applied in food and/or pharmaceutical products.

## 1. Introduction

Probiotics are becoming increasingly popular in food and pharmaceutical products due to their health benefits [1]. To provide probiotic benefits, probiotic products commonly contain at least 6 log cfu/g of viable cells at the time of consumption [2]. Some potential health benefits of probiotics have been the maintenance of the intestinal microbial balance, alleviation of lactose intolerance, serum cholesterol reduction, reduction of cancer risk, immune system stimulation and prevention of intestinal tract infections [3],[4],[5]. Nowadays, the most popular probiotic strains are *Lactobacillus* spp. and *Bifidobacterium* spp., which have been widely utilized for pharmaceutical purposes. However, probiotic cells are sensitive to processing conditions such as oxygen stress, freezing, drying and storage, as well as the harsh action of low pH and bile salt in the gastrointestinal tract. To cope with these problems, the development of processing techniques and formulations that enhance probiotic viability and the ability to deliver high numbers of viable cells to the target is greatly needed [6],[7],[8].

Encapsulation is defined as the encasement of the sensitive core ingredients such as solid, liquid or gaseous materials within a biopolymer shell that is able to release its contents at a controlled rate once stimulated by specific environments [9],[10]. Encapsulation, as one of the most useful methods, has been widely applied to protect the viability of probiotics [11],[12]. Using this technique, the probiotic cells are entrapped in capsules, which protect them from the detrimental factors of manufacturing processes and gastrointestinal tract conditions. Because of the many benefits offered by encapsulation, entrapped probiotic cells can be used to produce dairy products such as yoghurt, cheese and fermented milk products, as well as in pharmaceutical products such as tablets, capsules and sachets [13],[14]. The most common encapsulation techniques applied for probiotics have been spray drying, emulsion, coacervation and extrusion [15],[16],[17].

The spray-drying technique is a commonly used method of encapsulation in food industry, and it is also used for the encapsulation of live microorganisms [9],[18]. The principle of this technique is based on the atomization of a suspension of microbial cells in a polymeric solution into hot drying air, followed by the rapid evaporation of water. The microencapsulated product is then separated as a dry powder from the drying air via an outlet at a lower temperature [19],[20]. The technique, however, has also some major disadvantages for the encapsulation of probiotics in both food and pharmaceutical products because of a low survival rate of the bacteria during drying and low stability upon storage [21].

The emulsion technique involves the dispersion of the cell or polymer suspension (discontinuous phase) in an oil or organic phase (continuous phase). For the encapsulation of probiotic living cells by the emulsion technique, hydrocolloids such as alginate, pectin and carrageenan are used as encapsulating materials [22],[23]. The mixture of probiotic cell suspension and hydrocolloid wall material is incorporated into vegetable oil with consistent stirring to form a water-in-oil emulsion with the aid of emulsifier and surfactant [10]. The obtained capsules from this method have a small diameter, but it provides a large size range and shape. The emulsion technique yields a high survival rate of probiotic cells, and it is easy to scale up for large-scale production [10].

Coacervation is a modified emulsification technology. It has been attempted to encapsulate flavor oils, enzymes as well as microbial cells with the goal of enhancing their shelf life [24],[25]. Coacervation can achieve good encapsulation capacity and controlled liberation of the core material from the microspheres. It is especially useful for the encapsulation of probiotics, which are required to be released when exposed to higher pH in the large intestine [25]. However, the coacervation technique is limited in its usefulness as it requires higher costs and control of different critical conditions associated with composition and kinetics of reaction [26],[27].

Extrusion is a relatively simple technique and can be applied for encapsulation of food ingredients as well as probiotic cells. It involves preparing a hydrocolloid solution, adding microorganisms to it, and extruding the cell suspension through a syringe needle and placing drops of it into a hardening solution to form beads [28]. It is a cheap method with gentle operations, which is associated with minimal cell injury and relatively high viability of probiotic cells [9]. The extrusion method, however, is difficult for large-scale production because of its slow bead formation process. Even though it is considered a laborious technique, it is largely applied in research [9].

The selection of the wall material for encapsulation is a challenge. Biopolymer-based wall materials have been wildly used for the encapsulation of probiotic cells due to their non-toxicity, biodegradability, biocompatibility, as well as wide availability from natural resources [29],[30]. Among the biopolymeric encapsulating materials, alginate is extensively used [31],[32]. However, using only alginate has some disadvantages involving the porosity of the capsules [33] and capsule degradation in acidic environments [34]. In order to overcome the pitfalls of the alginate wall structure, co-encapsulation with other compounds such as proteins, prebiotics and starches has been reported [35],[36],[37].

Legume proteins have been increasingly applied in food industry as a replacement for animal-derived proteins because of their low cost and good functionality [38],[39]. Furthermore, the successful application of pea, chickpea and soy proteins in combination with polysaccharides as wall materials for probiotic delivery has been reported [40],[41]. Proteins used in conjunction with polymers can promote a synergistic effect on gelation providing further protection to probiotic cells and producing particles with an integrated structure [42],[43].

Bambara groundnut or *Vigna subterranea* (Leguminosae) is widely grown in the African continent, Brazil, as well as in Western Java and Southern Thailand [44],[45]. This commercially-interesting crop can grow on poor soils, possesses resistance to drought and pests, and has a long storage life [46],[47]. Concerning food and pharmaceutical purposes, protein from bambara groundnut can be used as a valuable supplement in the formulation of various types of functional food [48],[49]. Moreover, it has attractive characteristics to be a base hydrogel for the encapsulation of probiotics because of its biocompatibility, non-toxicity and ability to form gels [50],[51].

Few studies have investigated the potential of using legume protein isolates as co-encapsulating materials for probiotics. To obtain effective capsules, which increase encapsulation efficiency as well as viability of cells during exposure to the freeze-drying process and harsh conditions of the gastrointestinal tract, the optimization of the composition ratios of encapsulate materials is required. The classical approach for such an optimization is the one-variable-at-a-time technique, which requires a large number of experiments to describe the effects of individual factors. It is time consuming and cost non-effective [52]. Advantageously over the classical method, the response surface methodology (RSM) has been in use [53]. RSM is a statistical technique applied in developing, improving and optimizing processes in order to predict performance conditions with a minimum number of experiments [53].

This study aimed to find the optimal composition ratios of bambara groundnut protein isolate (BGPI) and alginate with the aid of RSM. It sought to maximize the encapsulation efficiency of *L. rhamnosus* GG. The cell viability of the probiotic in the developed capsules passing through freeze-drying and after exposure to simulated gastric conditions were evaluated. The ability of probiotic cells to be released from the encapsulated materials in simulated intestinal conditions and long-term storage were also investigated.

## 2. Materials and Methods

### 2.1. Bacteria strain and materials

*Lactobacillus rhamnosus* GG ATCC 53103 was purchased from the American Type Culture Collection (ATCC, USA). The frozen stock culture was reactivated twice in MRS broth (Difco, USA) and incubated under anaerobic conditions in an anaerobic jar containing the gas pak microbiology Anaerocult^®^ A (Merck, Germany) at 37 °C for 48 h. The cells were harvested by centrifugation at 3000 g for 10 min at 4 °C and washed twice before being re-suspended in normal saline solution (0.85% w/v NaCl). The cell concentration of 10 log cfu/ml served as the inoculum for the preparation of the encapsulated cells and the survival study. Alginic acid sodium salt (alginate), pepsin, bile salt and pancreatin were purchased from Sigma-Aldrich (USA), while calcium chloride, sodium chloride and sodium hydroxide were purchased from Ajax Finechem (Australia).

### 2.2. Preparation of bambara groundnut protein isolate

Bambara groundnut seeds were purchased from a local market in Hat Yai, Songkhla, Thailand. The sample was dehulled and ground using a grinding machine (Health Herb Products Co., Ltd., Thailand) to obtain a fine powder. The bambara groundnut protein isolate (BGPI) was prepared according to the methods described by Pastor-Cavada et al. [54], with a slight modification as follows. Twenty grams of powder were suspended in 100 ml of 2 g/l NaOH solution (pH 12). The mixture was stirred continuously for 2 h at room temperature (30 °C), followed by centrifugation at 8000 g for 30 min. The supernatant was collected and the pH was adjusted to 4.5 using 6 M HCl. The resultant precipitate was recovered by centrifugation at 8000 g for 30 min. The pellet was washed with 10 volumes of distilled water (pH 4.5), followed by centrifugation at 8000 g for 30 min.

The resulting pellet was freeze-dried in a freeze dryer (model FD-300 Airvac Engineering Pty Ltd., Dandenong, Australia). Regarding the freeze-drying conditions, the pellet was frozen at –40 °C for 2 h followed by 18 h of primary drying at –40 °C, 8 h of secondary drying at –10 °C, and finally, at 25 °C. The obtained dry powder was referred to as BGPI. BGPI was placed in a sealed polyethylene bag and stored at 4 °C until use. BGPI was subjected to proximate analyses at the Agro-industry Development Center for Export (ADCET), Faculty of Agro-industry, Prince of Songkla University, Hat Yai, Songkhla, Thailand. The crude ash, lipid, moisture and protein (%N × 6.25) contents of BGPI were determined according to the Association of Official Analytical Chemists [55] methods—923.03, 920.85, 925.10 and 920.87, respectively.

### 2.3. Encapsulation of *Lactobacillus rhamnosus* GG

#### 2.3.1. Experimental design and statistical analysis

*L. rhamnosus* GG were entrapped by BGPI or alginate with different concentrations. The preliminary results revealed that the encapsulation efficiency as well as the survival rates in acid condition and after freeze-drying of the bacterial cells was affected by the type of encapsulating materials and their concentrations. The optimal concentration ranges were 4–12% w/v for BGPI and 0.5–3% w/v for alginate. The capsules made of these individual materials with optimal ranges showed values of encapsulation efficiency of 85% and 89% for BGPI and alginate, respectively. Whereas, the survival rates in acid condition and after the freeze-drying process of the probiotic entrapped in BGPI were approximately 87% and 88%, respectively, and those in alginate were equally 85%.

In order to further improve the encapsulation efficiency as well as the survival rates, BGPI and alginate, as encapsulating matrix of probiotic cells, were combined in different ratios. The optimization process was performed using RSM with the central composite design (CCD) (Table 1). The ranges of concentrations of BGPI (A) and alginate (B) employed were as optimum, i.e., 4–12% w/v and 0.5–3% w/v, respectively. The CCD is a 2^k^ factorial design with star points and central points. The entrapment efficiency, survival rate in acid condition, and the rate after the freeze-drying process were selected as responses. The surface response plots were analyzed to reveal the effect of independent factors on the measured responses. The desirability function approach was used to develop the optimized formulation [56]. The general approach involves firstly the conversion of each response Y_i_ (i = 1, 2,..., m) into an individual desirability function *d_i_* that varies over a unit range, 0 ≤ *d_i_* ≤ 1, i.e., if response Yi is at its target goal, then *d_i_* = 1, and if it is completely within the undesirability range, *d_i_* = 0. Then, the designed variables were chosen to maximize the overall desirability as:

$$D={\left(d_{1}\times d_{2}\times \ldots d_{m}\right)}^{\left(1/m\right)}$$ (1)

where D is the desirability function and *m* is the number of responses.

The experimental design, data analysis, and optimization procedures were performed using the Design-Expert Version 9.0.3.1 software (Trial version 9.0.3.1; Stat-Ease, Minneapolis, USA; www.statease.com).

Regarding to the accuracy of the predicted model, the adequacy of the regression equation of the experimental data and the predicted values were determined using the *R^2^* and absolute average deviation (AAD) which was calculated by the following equation:

$$\mathrm{AAD}(\%)=\left\{\left[\sum_{i=0}^{p}\left(\left|y_{i,exp}–y_{i,cal}\right|/y_{\mathrm{i,exp}}\right)/p\right]\right\}\times 100$$ (2)

where y_i,exp_ and y_i,cal_ are the experimental and calculated responses, respectively, and *p* is the number of experimental runs.

#### 2.3.2. Preparation of encapsulated cells with the combination of BGPI and alginate

The probiotic capsules with various concentrations of the wall materials; BGPI (4–12% w/v) and alginate (0.5–3% w/v) were prepared according to the CCD's trials listed in Table 1. The encapsulation processes were performed by the extrusion technique following the methods described by Klemmer et al. [38], with a slight modification: the BGPI solution was prepared by dissolving BGPI powder in sterile distilled water (pH 8), followed by heating at 80 °C under constant mechanical stirring for 30 min. The solution was allowed to cool to room temperature, and the alginate powder was then added to the solution to the acquired concentrations. The BGPI-alginate solution was heated at 80 °C for 30 min with constant mechanical stirring. It was cooled down to room temperature and then the probiotic cells were added to a concentration of 10 log cfu/ml. The solution was transferred to a 10-ml syringe, and extruded through a 23G-needle into 30 ml of a sterilized 0.1 M CaCl_2_ solution to obtain the encapsulated capsules. The resulting capsules were magnetically stirred (250 rpm) for 30 min to allow them to harden, washed twice with sterile water, and freeze-dried. The fresh-prepared capsules were evaluated for encapsulation efficiency, whereas the freeze-dried ones were evaluated for the survival rate of the entrapped cells in acid condition and after the freeze-drying process using the enumeration method of free and encapsulated cells described in the following section. The formulation with the RSM's optimal result was selected for the study of morphology, survival rate, and release of encapsulated cells in both a simulated gastrointestinal fluid and storage conditions.

#### 2.3.3. Enumeration of free and encapsulated cells

The enumeration of free and encapsulated cells of *L. rhamnosus* GG was applied according to the following method: free cells were serially 10-time diluted with sterile normal saline solution, and the diluted solutions were plated on MRS agar. The colonies of *L. rhamnosus* GG were enumerated after incubation in anaerobic condition at 37 °C for 48 h. Regarding the enumeration of the encapsulated cells, the capsules were added into a 0.1 M phosphate buffer solution (pH 7.4), and the mixture was vigorously stirred for 30 min at room temperature to completely break the capsule and achieve the release of bacterial cells [7]. Appropriate dilutions were made in sterile normal saline solution, and the obtained solutions were plated on MRS agar. The colonies of *L. rhamnosus* GG were enumerated according to the method followed for the enumeration of free cells.

#### 2.3.4. Effect of encapsulation matrix on cell viability in pH 2.0 solution

The acid tolerance of free cells and the freeze-dried encapsulated cells was determined by the method suggested by Lee and Heo [57] as follows. The acid solution containing 0.2% w/v NaCl at pH 2 (adjusted by 0.1 N HCl) was prepared. A 1 ml of free cells and 0.1 g of encapsulated cells was added into test tubes containing 9 ml and 9.9 ml of acid solution, respectively. After incubation at 37 °C for 2 h, the acid solution was immediately decanted. The capsules were then washed twice with sterile normal saline solution to relieve acid stress. Then a 0.1 M phosphate buffer solution (pH 7.4) was added to the capsules, and the mixture was vigorously stirred for 30 min to completely release the bacterial cells from the capsules. The released bacterial cells were enumerated following the method described above.

#### 2.3.5. Effect of encapsulation matrix on cell viability after the freeze-drying process

The fresh capsule aliquots containing the probiotic were subjected to a subsequent freeze-drying process. The samples were distributed in Petri dishes and dried in a chamber-type freeze-dryer (model FD-300 Airvac Engineering Pty Ltd., Dandenong, Australia) at a condenser temperature of –40 °C for 20 h. The viable encapsulated cells before and after the freeze-drying process were enumerated, and the survival rate after the freeze-drying process was calculated.

### 2.4. Morphological characterization

The surface and inner morphology of the freeze-dried capsules were determined by scanning electron microscopy (SEM). The capsules were mounted on stubs and coated with gold in a sputter coater before being examined under SEM (JSM-5800LV, JEOL, Japan). Fifteen capsule diameters were measured to obtain the average measurement values.

### 2.5. Survival of free and encapsulated cells in simulated gastric fluid

The survival of free and encapsulated cells was determined in simulated gastric fluid (SGF). The SGF was composed of 3 g/l pepsin and 9 g/l sodium chloride in sterile distilled water, the pH of which was adjusted to 2.0 with 1 M HCl; it was also sterilized by filtration [58]. In the case of free cells, 1 ml of *L. rhamnosus* GG was added to 9 ml of SGF. The final SGF solution contained the probiotic cells of ∼10^9^ cfu/ml. After incubation at 37 °C, the sample aliquots were removed at 0, 30, 60, 120 and 180 min for inspection. Bacterial survival was measured according to the method described above. In the case of encapsulated cells, 0.1 g of freeze-dried capsules was added to 9.9 ml of SGF and incubated for 0, 30, 60 120 and 180 min at 37 °C. For each incubation time, the capsules were sampled and rinsed twice with normal saline solution before the determination of viable cells according to the method described above.

### 2.6. Release of Lactobacillus rhamnosus GG cells from capsules in simulated intestinal fluid

The ability of the encapsulated probiotic cells to be released from the designed capsules in simulated intestinal fluid (SIF) was tested. The SIF was composed of 1 g/l pancreatin and 3 g/l bile salts in 12.5 g/l of NaHCO_3_, and its pH was adjusted to 7.0 with 1 M NaOH [59]. The solution was sterilized by filtration. The survival of free cells and the profile of their release from the freeze-dried capsules without SGF treatment were investigated. The free cells and capsules were incubated in SIF in a similar fashion as in the SGF test. At determined time points (0, 30, 60, 120, 240 min), 100 µl of the liquid phase (without visible capsules) was withdrawn from the tubes and immediately used for the enumeration of viable cells.

### 2.7. Survival of free and encapsulated cells during storage

The freeze-dried encapsulated probiotic cells (400 mg) were manually packed into a gelatin capsule No. 1. The capsules were placed in an air-tight amber glass bottle containing silica gel desiccant packed down tightly with a wad of cotton wool. The freeze-dried free cells were stored in a 1-ml sealed glass ampule. The samples were kept at both 4 °C in a refrigerator and room temperature (30 °C) for 6 months. They were enumerated for the viable cells monthly during storage.

### 2.8. Statistical analysis

The statistical optimization was performed using the Design-Expert Version 9.0.3.1 software (Trial version 9.0.3.1; Stat-Ease, Minneapolis, USA; www.statease.com). All of the experiments in this study were conducted in triplicates for each treatment. All measured data were expressed as mean ± standard deviation (SD). A Student's t-test was used to determine statistical differences (*p* < 0.05) in all experiments.

## 3. Results and Discussion

### 3.1. Chemical composition of bambara groundnut protein isolate

The bambara groundnut protein isolate (BGPI) was extracted from dry seed powder under alkaline condition. The yield was 25.70% w/w, which was calculated from the total weight of the powder. The proximate chemical composition of BGPI included 87.62% protein, 2.16% fat, 1.54% ash, 5.44% carbohydrate and 3.24% moisture. Our findings were in line with those of a previous study, in which the protein, fat, ash, carbohydrate and moisture proportions were 85.21%, 3.24%, 3.11%, 5.48% and 3.16%, respectively [60].

### 3.2. Response surface design

#### 3.2.1. Optimization of BGPI and alginate concentrations for the encapsulation of *Lactobacillus rhamnosus* GG

RSM was applied to determine the effect of BGPI (A) and alginate (B) concentrations on the encapsulation efficiency, as well as survival rate of *L. rhamnosus* GG in both acid condition and after the freeze-drying process. The results of the experiments performed according to the CCD of the variables along with the responses are given in Table 1. Meanwhile, the regression coefficients obtained for the different responses and the ANOVA results for the model responses are presented in Table 2.

For goodness of fit, it was suggested that the *R*^2^ value should be greater than 0.80 [61]. The *R*^2^ values for the models predicting the responses, i.e., encapsulation efficiency, viability in acid condition and after the freeze-drying process, were 0.9867, 0.9803 and 0.9773, respectively, indicating the model was significant and implying the adequacy of the applied regression model. The lack of fit for all fitted models was found to be not significant (*P* > 0.05). Myers and Montgomery [62] suggested that the lack of fit measures the failure of the model to represent data in the experimental domain at points which are not included in the regression. Thus, it is presumably ensured that the selected model can be used for the simulation and optimization of variables in achieving the desired purposes.

**Table 1.** Treatment combinations of encapsulation agents (bambara groundnut protein isolate, BGPI with alginate) for *Lactobacillus rhamnosus* GG according to the central composite design with experimental and predicted values.

Trial no.	Factor (%)		Responses					
	BGPI	Alginate	Encapsulation efficiency (%)		Survival rate (%) in acid condition		Survival rate (%) after freeze-drying	
			Experimental	Predicted	Experimental	Predicted	Experimental	Predicted
1	–1 (4)	–1 (0.5)	84.94	84.77	83.84	83.38	83.60	83.34
2	1 (12)	–1 (0.5)	87.69	88.61	89.01	89.05	88.65	89.10
3	–1 (4)	1 (3)	88.93	88.67	86.77	87.14	87.08	87.43
4	1 (12)	1 (3)	87.97	88.80	87.34	88.21	87.78	88.84
5	–1.4142 (2.34)	0 (1.75)	85.95	86.38	85.14	85.28	83.97	84.06
6	1.4142 (13.65)	0 (1.75)	90.27	89.21	90.61	90.06	90.02	89.16
7	0 (8)	–1.4142 (0)	86.58	86.40	84.81	85.38	86.36	86.56
8	0 (8)	1.4142 (3.51)	89.34	89.16	88.05	87.33	89.93	89.16
9	0 (8)	0 (1.75)	96.68	97.17	96.51	95.38	95.04	94.98
10	0 (8)	0 (1.75)	96.79	97.17	95.27	95.38	94.10	94.98
11	0 (8)	0 (1.75)	97.21	97.17	94.34	95.38	95.73	94.98
12	0 (8)	0 (1.75)	97.44	97.17	94.88	95.38	95.68	94.98
13	0 (8)	0 (1.75)	97.71	97.17	95.89	95.38	94.33	94.98

**Figure 1.**
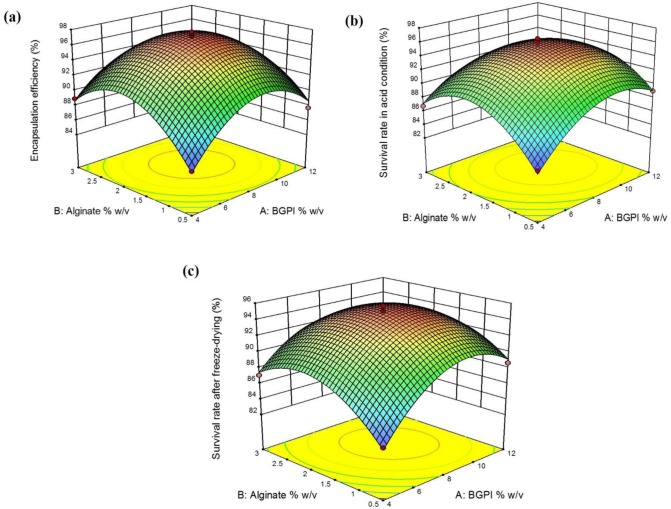
Response surface plots of interaction between bambara groundnut protein isolate (BGPI) and alginate on encapsulation of *Lactobacillus rhamnosus* GG: (a) encapsulation efficiency, (b) survival rate in acid condition (pH 2.0, 37 °C), and (c) survival rate after the freeze-drying process.

In the probiotic encapsulation experiment, the response surface plots (Figure 1a) suggested that the optimal concentrations of BGPI and alginate were 8% w/v and 2% w/v, respectively. The interaction effect between the two variables showed a negative effect on the response. The regression equation obtained for apparent encapsulation efficiency as response variable is given below:

Y1=63.0579+5.2635A+12.9787B–0.1856AB–0.2932A2–3.0500B2 (3)

In the response plot for variables on the survival rate of *L. rhamnosus* GG in acid condition (Figure 1b), as was the case for the apparent encapsulation efficiency, the increase in BGPI concentrations showed a gradual rise and then a slight drop in the survival rate of cells in acid condition. It was noticed that both higher and lower mean concentrations of BGPI and alginate had a detrimental effect on the viability of *L. rhamnosus* GG in acid condition. A negative interaction effect between the BGPI and alginate concentrations was also observed. The regression equation for the response survival rate of *L. rhamnosus* GG in acid condition is given below:

Y2=63.3851+4.6779A+12.6764B–0.2299AB–0.2409A2–2.9291B2 (4)

Figure 1c shows the effect of BGPI and alginate concentrations on the viability of *L. rhamnosus* GG after the freeze-drying process. It was observed that the viability of the freeze-dried cells steadily increased with the increase in the concentration of BGPI and alginate up to their mean values, but it decreased slightly beyond these concentrations. In this case also, the interaction effect was found to be negative. The regression equation for the response survival rate of *L. rhamnosus* GG after the freeze-drying process is given below:

Y3=63.1793+5.0162A+10.5994B–0.2176AB–0.2617A2–2.3120B2 (5)

Both BGPI and alginate showed a significant quadratic effect. All of these significant interaction terms were also associated with negative regression coefficients indicating that when one increases, the other decreases, and vice versa – this will contribute to an increase in every response.

**Table 2.** Analysis of variance (ANOVA) for evaluation of second-order response surface model.

Reponses	Source	Sum of square	Degree of freedom	Mean square	*F* value	*P* value
Encapsulation Efficiency (%)	Regression	294.85	5	58.97	104.25	0.0001
	Residual	3.96	7	0.57		
	Lack of fit	3.20	3	1.07	5.64	0.0641
	Pure error	0.76	4	0.19		
	Total	298.81	12			
Survival rate (%) in acid condition	Regression	253.03	5	50.61	69.62	0.0001
	Residual	5.09	7	0.73		
	Lack of fit	2.22	3	0.74	1.04	0.4669
	Pure error	2.86	4	0.72		
	Total	258.12	12			
Survival rate (%) after freeze-drying	Regression	226.26	5	45.25	60.39	0.0001
	Residual	5.25	7	0.75		
	Lack of fit	2.98	3	0.99	1.75	0.2948
	Pure error	2.27	4	0.57		
	Total	231.51	12			

**Figure 2.**
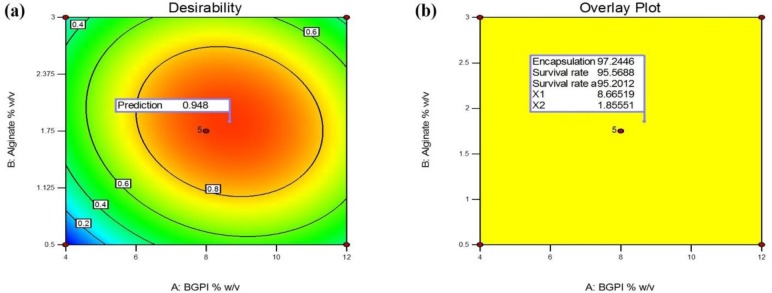
Desirability plots: (a) indicating desirable regression ranges and the overlay plot, and (b) indicating the region of optimal process variable settings (BGPI: bambara groundnut protein isolate).

Optimization was based on the maximization of the encapsulation efficiency, and survival rate in acid condition and the rate after the freeze-drying process. The optimal condition was found to be 8.66% w/v for BGPI and 1.85% w/v for alginate. Under these conditions, the desirability function was 0.948, close to the maximum of 1, which is acceptable taking into account the fact that the responses were simultaneously optimized (Figure 2).

#### 3.2.2 Verification of the predictive model

The model was validated by conducting random experiments within the experimental area. The predictability of the model for all of the responses was determined by comparing the experimented values with the values predicted by the model. To determine the validity of the statistical model, the experimental values of all responses were compared to the predicted values by calculating the absolute average deviation (AAD). The calculated AAD values for these quadratic models were 0.48%, 0.59% and 0.59% for encapsulation efficiency, survival rate in acid condition and survival rate after the freeze-drying, respectively. These results indicated that the model equations were accurate and highly reliable.

The encapsulation of *L. rhamnosus* GG with a BGPI-alginate matrix at the optimal concentration suggested by the model was applied. Under the optimized concentrations of encapsulating materials (8.66% w/v BGPI and 1.85% w/v alginate), the predicted encapsulation efficiency, survival rate in acid condition and survival rate after the freeze-drying process were 97.24%, 95.56% and 95.20%, respectively. Whereas, the experimented values showed that all three responses were reliable—97.07% (9.93 log encapsulation) for encapsulation efficiency, 95.18% (0.49 log reduction) for survival rate in acid condition and 94.85% (0.53 log reduction) for survival rate after the freeze-drying process. The results confirmed the validity of the model and showed that the experimental values were close to the predicted ones. The absolute deviation from the mean were 0.17%, 0.39% and 0.36% for encapsulation efficiency, survival rate in acid condition and survival rate after the freeze-drying process, respectively. In addition, the satisfactory correlation between the experimental and predicted values confirmed that the response model had adequately reflected the optimization process.

### 3.3. Capsule morphology and size

**Figure 3.**
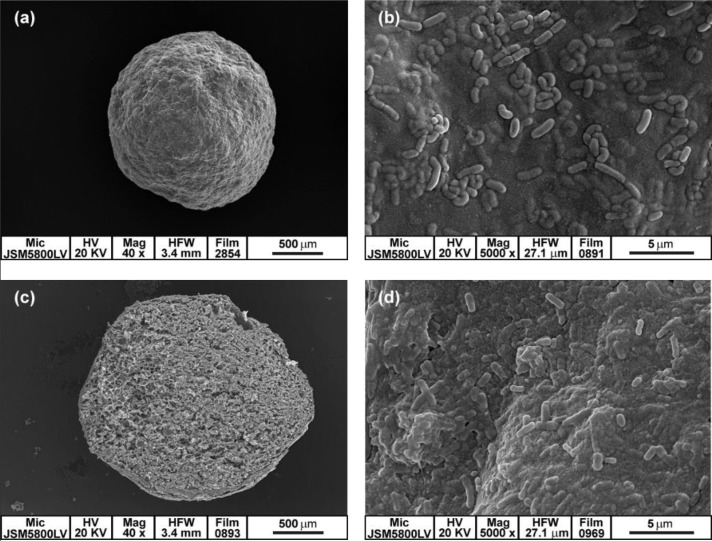
Scanning electron micrograph of *Lactobacillus rhamnosus* GG cells entrapped in 8.66% w/v bambara groundnut protein isolate (BGPI) combined with 1.85% w/v alginate capsules: (a) freeze-dried capsule (40×), (b) capsule outer surface (5000×), (c) capsule cross section (40×), and (d) capsule internal surface (5000×).

The morphology and size of the BGPI-alginate capsules were investigated using SEM (Figure 3). The capsules were spherical in shape and had a rough surface without cracking. The *L. rhamnosus* GG cells were covered with a thin matrix and presented randomly on the surface (Figure 3a and 3b). The cross section of the capsule revealed a mesh matrix structure, and bacteria cells were entrapped within the capsules (Figure 3c and 3d). Apparently, the capsules were dense and compact having a size range of 1.25 ± 0.37 mm. The encapsulation made of the combination of protein or starch and alginate led to a greater compaction of the capsule structure [38],[63]. Thus, reducing capsule wall leakage and increasing protection to the entrapped probiotic cells were expected.

### 3.4. Survival of free and encapsulated probiotic cells in simulated gastric fluid

**Figure 4.**
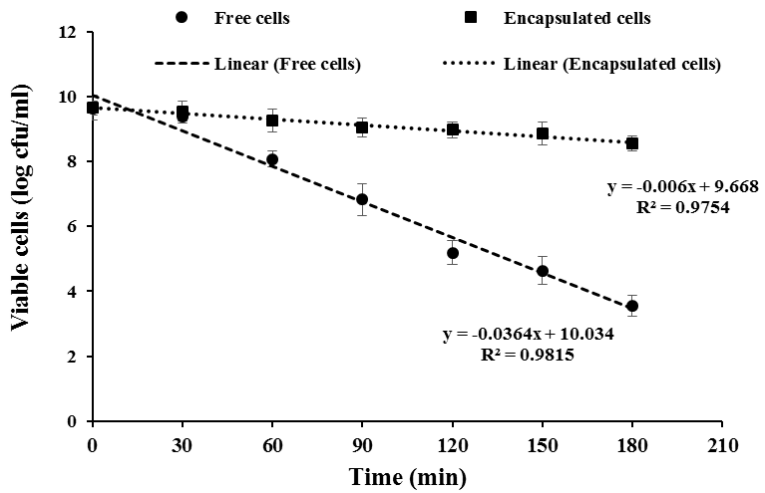
Survival of free and encapsulated cells of *Lactobacillus rhamnosus* GG in capsules containing 8.66% w/v bambara groundnut protein isolate (BGPI) combined with 1.85% w/v alginate during exposure to simulated gastric fluid (pH 2.0, 37 °C).

The survival of *L. rhamnosus* GG in simulated gastric fluid (SGF) (pH 2.0) at 37 °C was studied for both free and BGPI alginate-entrapped cells over a 3-hour period (Figure 4). As seen in Figure 4, the entrapped *L. rhamnosus* GG cells experienced significant (*P* < 0.05) protection from SGF condition with the end-point cell reduction values of ∼1.1 log cfu/ml. In the case of free cells, a significant log reduction (∼1.6 log cfu/ml) in cell viability was observed within the first 60 min. It continued to decline, yielding an end-point cell-reduction value of 6.1 log cfu/ml after 180 min. Overall, the entrapped *L. rhamnosus* GG showed a significantly (*P* < 0.05) higher survival than free cells during the SGF-challenge study. These findings were in agreement with the results reported by Chitprasert et al. [64] and Zhang et al. [65]. They showed that the probiotic cells entrapped in soy protein isolate-alginate capsules exhibited a significantly greater number of viable cells than free cells when exposed to SGF.

A BGPI alginate-Ca^2+^ network probably developed, entrapped the cells, and helped maintain capsule integrity. The BGPI-alginate capsules are presumed to be formed through a cross-linking process between alginate and calcium ions (Ca^2+^). The formation of egg-box-like junction zones within alginate capsule likely follows a similar mechanism as proposed by Sheu and Marshall [66]. They described a mechanism for crosslinking of alginate with Ca^2+^ produced using an external gelation process. Immediately after the extrusion of alginate-probiotic mixture into the cross-linking solution, gelation occurred at the periphery of the aqueous droplet, forming a solid Ca^2+^-alginate capsule. A diffusion front of Ca^2+^ ions was proposed to form and it diffused into the interior of the capsule until all of the Na^+^ ions have been replaced with Ca^2+^ ions, leading to the formation of a porous microstructure [67],[68]. Furthermore, BGPI may fill the vacant space within the porous matrix leading to further densification which allowed less probiotic diffused out of the capsule. And, it could also act as a physical barrier to protect the cells from SGF [38].

### 3.5. Release of encapsulated probiotic cells in simulated intestinal fluid

**Figure 5.**
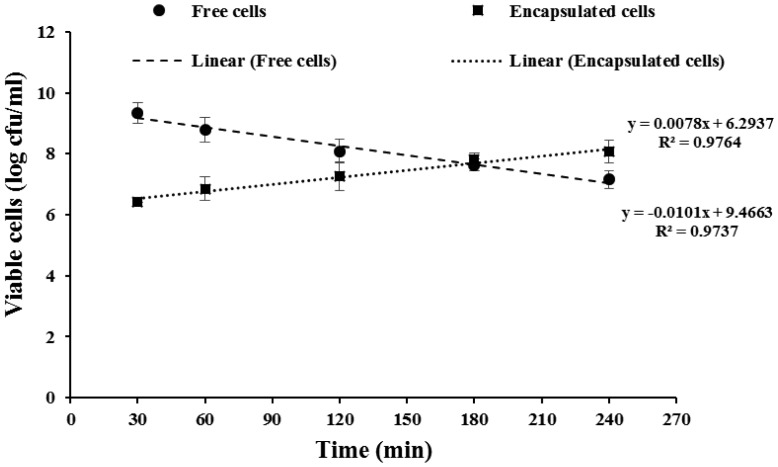
Release of *Lactobacillus rhamnosus* GG from capsules containing 8.66% w/v bambara groundnut protein isolate (BGPI) combined with 1.85% w/v alginate, and survival of free cells during exposure to simulated intestinal fluid (pH 7.0, 37 °C).

The release of entrapped *L. rhamnosus* GG cells from the BGPI-alginate capsules immersed in simulated intestinal fluid (SIF) (pH 7.0) incubated at 37 °C for 4 h was studied (Figure 5). After 30 min of exposure, ∼6.4 log cfu/ml of viable cells were released and continued to reach ∼8.0 log cfu/ml over a 4-hour period. The release of the probiotic was comparable to that reported by Wang et al. [69].

They prepared a pea protein isolate-alginate and found that the entrapped probiotic cells were released in SIF at a rate of 6 to 7 log cfu/ml within 3 h. While in the case of free cells, the viable cells in SIF decreased in number slightly from about 9.34 log cfu/ml at the beginning to 7.1 log cfu/ml after 4 h; entrapped cells increased from 6.4 log cfu/ml to 8 log cfu/ml. This phenomenon may be due to the effect of the bile salt ingredients in SIF, resulting in a poorer survival of bacterial cells [70]. The release of probiotic cells from the capsules in SIF may be explained by the increase in degradation of the probiotic capsules owing to the presence of NaHCO_3_ and pancreatin. Smidsrod and Skjak-Braek [70] explained that Na^+^ from NaHCO_3_ could act as an anti-gelling agent causing disruption in the alginate-calcium network whereas protease enzyme could partially hydrolyze BGPI leading to the breakdown of the capsule. And, the degradation of the capsule walls was followed by the release of the entrapped probiotic cells into the medium. Similar probiotic release behavior involving *Lactobacillus casei* ATCC 393 entrapped in pea protein isolate-alginate was reported by Xu et al. [71]. They suggested that the developed encapsulated formulations have potential for the targeted delivery of probiotics to the lower gastrointestinal tract, where they have the potential to colonize, thus, expressing health benefits to the host.

### 3.6. Storage stability of free and encapsulated probiotic cells

**Figure 6.**
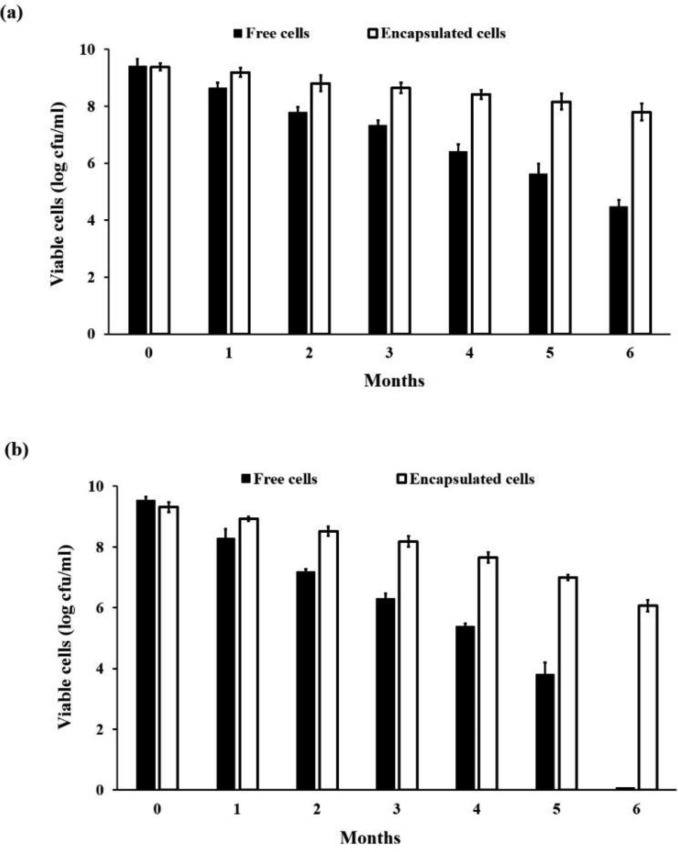
Survival of free and encapsulated *Lactobacillus rhamnosus* GG cells in capsules containing 8.66% w/v bambara groundnut protein isolate (BGPI) combined with 1.85% w/v alginate during a 6-month storage period at (a) 4 °C and (b) 30 °C.

The storage stability of the free and encapsulated *L. rhamnosus* GG is shown in Figure 6. In both storage temperatures, greater viability of encapsulated cells showed higher storage stability compared to that of free cells. At 4 °C, the viability of the free cells significantly dropped from 9.41 log cfu/ml to 4.47 log cfu/ml after 6 months. While the survival numbers of the encapsulated cells was slightly reduced from 9.38 log cfu/ml to 7.79 log cfu/ml after 6 months (Figure 6a). During storage at 30 °C, the number of viable free cells (9.54 log cfu/ml) decreased continuously and could not be detected after 6 months (Figure 6b). The number of viable cells from the encapsulated capsules, in contrast, fell from 9.31 log cfu/ml to 8.18 log cfu/ml after 3 months, and their numbers remained at approximately 6.07 log cfu/ml at 6 months.

Many studies have also showed that the survival rate of lyophilized free and encapsulated cells was higher at refrigeration temperature compared to room temperature [71],[72],[73]. This difference can be attributed to the reduction in the rate of fatty acid oxidation on the cell membrane [74]. On the other hand, the elevated storage temperatures result in increased rates of detrimental chemical reactions; for instance, the fatty acid oxidation at room temperature leads to a lower survival rate compared with that at colder storage temperatures. In this study, the higher survival rate of the encapsulated cells compared to that of free cells stored in both temperatures indicated that the BGPI-alginate capsules act as a protective barrier against the detrimental effect of oxygen on the cells during storage. Hugo et al. [75] reported a similar finding for probiotic cells encapsulated in a soy protein isolate with a calcium chloride matrix. They suggested that sulfur-containing amino acids present in soybean proteins could contribute to the maintenance of low redox potentials and also act as oxygen scavengers leading to an improved cell survival during storage. Thus, it is possible to hypothesize that sulfur-containing amino acids present in BGPI may play a protective role similar to those found in soybeans.

## 4. Conclusion

The present study reports the successful development of BGPI-alginate encapsulated capsules containing *L. rhamnosus* GG. The optimization concentration of the encapsulating agents was achieved using RSM. The optimal formulation of *L. rhamnosus* GG-loaded BGPI-alginate capsules were prepared using 8.66% w/v BGPI and 1.85% w/v alginate that yielded capsules with a greater cell-encapsulation efficiency, significantly higher survival rates in both acid condition and after the freeze-drying process. The capsules were spherical in shape and the bacteria were entrapped on the surface as well as inside the capsule. The entrapped cells were protected when exposed to simulated gastric fluid, and efficiently released in simulated intestinal fluid. The storage stability data demonstrated that the BGPI-alginate wall material provided better protection, significantly extending the survival of *L. rhamnosus* GG relative to free cells over a 6-month period. Thus, the bambara groundnut protein isolate promises to be a strong co-encapsulating material candidate for probiotic delivery systems.
